# TV listening and hearing aids

**DOI:** 10.1371/journal.pone.0200083

**Published:** 2018-06-29

**Authors:** Olaf Strelcyk, Gurjit Singh

**Affiliations:** 1 Sonova U.S. Corporate Services, Warrenville, Illinois, United States of America; 2 Phonak Canada Ltd, Mississauga, Ontario, Canada; 3 Department of Speech-Language Pathology, University of Toronto, Toronto, Ontario, Canada; 4 Toronto Rehabilitation Institute – University Health Network, Toronto, Ontario, Canada; 5 Department of Psychology, Ryerson University, Toronto, Ontario, Canada; Center for Healthy Start Initiative, NIGERIA

## Abstract

TV listening plays a large role in the lives of hearing-impaired (HI) individuals. Yet, few studies have examined TV listening in this group. In this paper, we report the findings of an online survey on TV listening conducted among HI individuals with and without hearing aids (HAs) in the United States in 2015. The research investigated if and in what form TV listening experiences of unaided and aided HI individuals might differ with regard to their viewing habits, difficulties they experience, and compensation strategies they employ. 515 HI people of ages 50+ years participated, 260 of whom owned HAs. HA users reported that they watched TV or video on average for 6 hours 10 min per day, 57 minutes longer than the duration reported by non-HA owners. Furthermore, HA users indicated fewer difficulties when watching TV than non-HA owners, suggesting that HA usage alleviated difficulties with TV listening. Nevertheless, the most frequent problems were still encountered by more than 39% of the HA users. Difficulties increased with greater self-reported unaided hearing disability, and female participants indicated more problems than male participants. Finally, those with carpeted floors reported fewer difficulties than those without carpets. The most frequently used compensation strategies were changing TV or HA volumes and using closed captioning. Only few HA users used audio streaming accessories. Given the exploratory nature of this study, further research is needed to inform interventions and improve the TV listening experiences of HI viewers.

## Introduction

Watching and listening to media (e.g., broadcast television (TV), digital videos, DVDs, etc.) plays a large role in the lives of many today. Consumer research company Nielsen [1] reports that adults ages 18+ years in the United States spent 5 hours 22 minutes per day watching media in 2017. Furthermore, usage increases with age. For example, adults ages 50+ years watched 7 hours per day. Using ecological momentary assessments, Hasan et al. [2] measured the listening activities of 19 hearing-aid (HA) users (ages 65+ years) and found that the two most frequent listening activities were conversations and listening to media, 33% and 31% of the time, respectively. Two recent studies, one with 28 HA users (ages 64+ years) [3] and another with 29 non-HA users with mild hearing losses (ages 55+ years) [4] found media-listening percentages of 35% and 21%, respectively (the 21% comprised speech listening but did not include listening to non-speech sounds such as music). Similar to conversations, media listening can be challenging. The Voice of the Listener & Viewer, a British broadcasting consumer group, reported that the most common complaint they received was difficulty understanding speech in TV programs [5]. The British Broadcasting Corporation (BBC) has been aware of the problem for many years [6–8] and published a best-practice guidance for program makers in 2011 [9] in an effort to improve TV speech intelligibility by encouraging good practices early in the production chain.

Given the high prevalence of media listening, surprisingly few studies have investigated unaided and/or aided media listening by the hearing impaired (HI). Consumer satisfaction reports [10–12] indicate a trend for increased satisfaction with HAs when listening to TV: the percentage of satisfied HA customers increased from <70% in the years up to the year 2000 to 80% in 2008. On the other hand, Gordon-Salant and Callahan [13] measured the effects of HAs and/or closed captioning (CC) on word recognition by 15 HA users with mild to profound losses (ages 59+ years). They used equalized lists of audio-visual sentences compiled from three TV programs (news, game shows, and dramas), which were presented via a flat-screen TV at a level of 60 dBA. Although higher recognition scores were observed with CC than without, HA use did not improve performances significantly. However, recognition scores trended higher in the HA than in the unaided condition, and thus, the lack of a significant HA effect may have been due to low statistical power rather than the absence of HA benefit per se. 13 of the 15 study participants reported they never used CC when watching TV. Hence, Gordon-Salant and Callahan suggested raising awareness about CC among HI viewers as an effective means to ameliorate problems with understanding speech on TV. The low CC usage in their sample may not be representative of broader populations, as a recent study [14] among people with disabilities in Germany found that 45% of the 65 HI participants used CC at least sometimes (79% of these were HA users).

Shirley [15] examined the benefits of presenting TV dialogs to HI listeners via a dedicated center loudspeaker compared with down-mixed stereo presentation via two loudspeakers. Those who stated that they typically wore hearing aids when watching TV performed the tests aided, while the other participants performed the tests unaided. Shirley found that a dedicated center loudspeaker significantly improved speech clarity compared with stereo presentation. He attributed the observed benefit to reduced cross-talk artifacts in the case of the dedicated center speaker. An alternative explanation for the benefit might be an increased spatial release from masking in the dedicated-center condition [16,17]. Apart from a dedicated center loudspeaker, some recent studies have explored the benefits of providing “clean audio,” i.e., dedicated audio services with higher dialog intelligibility, to HI viewers [18,19]. Although the results are promising, it remains unclear if and when such audio services will be made available to viewers.

Williger and Hannemann [20] reported the findings of a TV-listening online survey of 83 hearing-impaired HA users of ages 60+ years. They found that most of the participants (88%) watched TV in the living room and had flat-screen TVs (93%) with loudspeakers in or at the TV (93%) as opposed to in the room. 51% of the participants indicated they watched TV in the company of a normal-hearing (NH) person, 39% watched TV by themselves, 8% watched in the company of a HI person, and 2% watched TV together with several people. The participants experienced difficulties most frequently with movies and talk shows and least frequently with newscasts and documentaries, which is consistent with another recent survey [19]. Furthermore, difficulties increased with increasing degree of self-reported unaided hearing disability. Regarding adaptation strategies, 78% of the participants reported manipulating the volume of the TV, and 49% changed the volume of the HAs. 48% specified that they or someone else had adjusted the acoustic settings of the TV (such as low-frequency vs. high-frequency balance) according to their needs, and 30% indicated that they used a specific TV-listening program in their HAs. The authors stressed that further studies were needed to inform potential interventions for HA users.

Indeed, TV listening by the hearing impaired appears to be an understudied topic. This may be due to a notion that TV listening is ‘just another’ speech-in-noise problem that does not warrant further study beyond the vast body of research on speech-in-noise communication. However, TV listening critically differs in several respects from conversational speech-in-noise scenarios. While interference from background music, noise, and sound effects had traditionally been considered the main factor limiting TV speech intelligibility [6,7,21], more recent studies have shown that audience complaints about poor TV speech intelligibility can be attributed to several additional factors. Other detrimental factors are foreign accents and dialects, mumbling talkers and poor diction, fast speech, and speech in reverberation [5,8,19,22]. TV exposes viewers to a large diversity and fast-paced changes in talker voices, accents, speaking rates, and acoustic scenes. Importantly, for most viewers, this diversity may be larger than what they experience in their everyday listening situations. Exacerbating the issue, age-related hearing impairment can reduce the ability to compensate for changing talkers, speaking rates [23], and novel accents [24]. Thus, it is likely the case that the greater and more fast-paced acoustic diversity featured on TV poses greater challenges for HI than NH listeners. In addition, the vast majority of modern flat-screen TVs have small built-in loudspeakers mounted either down-firing or rear-firing, i.e., they are directed at the tabletop below or wall behind the TV. Thus, compared to a situation with a loudspeaker or talker directly facing the listener (as is common in laboratory studies), reverberation is enhanced, and the direct-to-reverberation ratio is often reduced to such an extent that listeners sit outside the critical distance in the reverberant sound field [25,26], which very likely compounds HI listeners’ difficulties with understanding speech on TV [27–30]. Also, dynamic range compression is enabled by default on most TVs, and thus, TV audio is compressed at least twice for HA users: by the TV and subsequently by their HAs. This could result in over-compression and potentially degraded speech intelligibility [31,32]. Finally, watching TV is an audiovisual process. Many HI individuals rely on lip reading. However, this may be easier in real-world conversational settings than on TV where the talker often may not be visible on screen or may not face the camera [15]. On the other hand, TV has one advantage compared with real-world listening: it provides CC. Overall, the extant literature would suggest that TV listening is not just a speech-in-noise task. Many distinctive features of TV listening, and their consequences, especially for HI audiences, have not been fully explored and deserve further study.

Regarding amplification, to our knowledge, only Gordon-Salant and Callahan [13] have previously assessed effects of HA usage on TV listening. However, their study exclusively focused on TV speech intelligibility and involved a relatively small sample of 15 HI participants. In contrast, here we report the results of a comprehensive online survey on TV/media listening conducted on a large sample of HI individuals with and without HAs. We compared the responses of both groups to answer the research question if and in what form their TV listening experiences might differ with regard to their viewing habits, difficulties they experience, and compensation strategies they employ.

## Methods

### Procedures

Participants were recruited in the United States in April to July 2015, using Amazon Mechanical Turk (MTurk). MTurk comprises anonymous workers who complete tasks online in exchange for monetary payment. Data obtained from MTurk participants have been found to be at least as reliable as those obtained via traditional methods [33–36] including research in audiology [37]. All participants were asked to meet the following criteria: had to have a self-reported hearing impairment, be 50 years of age or older, and had to have learned English before the age of 10. Furthermore, they had to live in the United States and watch electronic media (e.g., TV, movies, online videos) for at least 3 hours per week. The surveys were created using SurveyMonkey online survey software (www.surveymonkey.com) and took, on average, 16 minutes to complete. Participants were paid $1.00 USD for their time. The study was approved by the Institutional Research Ethics Board of the University Health Network in Toronto.

### Materials

Participants were asked to complete four questionnaires. In addition to the main questionnaire about TV/media listening, the survey included questionnaires about demographics, perceived hearing handicap when unaided, and expectations for HA ownership or satisfaction with HAs for the non-HA owners and HA owners, respectively.

The first questionnaire about demographics consisted of seven items and inquired about gender, age, bilateral vs. unilateral HA usage, the brand(s) of HAs currently used (participants could select from a list of 35 HA brands), and brand(s) of PSAPs currently used in case the participants did not find their HA brand in the previous list (participants could select from a list of 52 PSAP brands). Two more questions probed the participants’ eyesight, asking them to rate their eyesight using both eyes and the difficulty experienced when reading news tickers displayed on the bottom half of the TV screen (see S1 Dataset for the exact wording of the questionnaire).

The TV/media listening questionnaire consisted of 31 items (see S1 Appendix). Although the questionnaire explicitly inquired about TV and video listening, including video on demand, DVDs, streaming video, etc., we will be referring to TV listening in the following for sake of brevity. The items inquired about the daily time spent watching TV/media, viewing locations, viewing devices and audio systems, self-rated importance of hearing well when watching TV, usage of the remote control to change volume, satisfaction with self- and other-adjusted TV loudness, level of difficulty with understanding speech on TV, strategies to compensate for difficulties with understanding speech, usage of CC, usage of HAs, and satisfaction with HAs when watching TV (only included in the questionnaire for the HA owners). In addition, TV item 23 was a closed-ended question that inquired about problems encountered when watching TV. Participants were asked to select all responses that applied from a list of response options. The following item, TV24, was an open-ended question asking the participants to indicate any other problems that they encountered when watching TV. After 264 participants had responded to the survey, a preliminary analysis of their responses to the open-ended item TV24 revealed three responses that were mentioned repeatedly but had not been anticipated in the closed response set of item TV23. Therefore, for the remaining 251 participants, these three responses were added as additional response options to item TV23. Similarly, item TV19 about compensation strategies also offered an open response option, which allowed the participants to supply their own strategies. Again, a preliminary analysis of the responses from the first batch of participants revealed two compensation strategies that had not been anticipated. These were added as response options for the remaining participants. The TV/media listening questionnaire also included a question about specific films, broadcasts, or videos with poor intelligibility (TV25) as well as six additional items about radio and music listening (TV26 to TV31). However, these items are not discussed in the present article.

The third questionnaire was the Hearing Handicap Inventory for Adults (HHIA) [38], which is a 25-item measure of social and emotional consequences of hearing impairment with higher scores indicating greater hearing handicap. The HA owners were asked to answer the way they heard *without* HAs. Thus, the HHIA measured the participants’ unaided hearing handicaps.

The fourth questionnaire differed for the non-HA and HA owners. The non-HA owners answered the Expected Consequences of Hearing Aid Ownership questionnaire (ECHO) [39]. It measured expectations about HAs by asking individuals to rate 15 items on positive effects of HAs, their service and cost, their negative features, and effects on personal image. An additional item asked individuals to “rate the severity of [their] hearing impairment” with the response options “None,” “Mild,” “Moderate,” and “Severe” for each ear. In the following, we refer to the rating for the worse ear as ‘self-reported degree of HI’. The HA owners answered the Satisfaction with Amplification in Daily Life questionnaire (SADL) [40]. SADL measured satisfaction with HAs using 15 items in the same categories as ECHO, i.e., positive effects, service and cost, etc. In addition, it included five questions about types of HAs, HA experience, daily HA usage, and severity of hearing impairment. Past research has observed the SADL to be a reliable [40] and valid measure of hearing aid satisfaction at 1 year post-fitting [41].

### Initial data filtering

A total of 710 individuals responded to the surveys. The datasets from 515 respondents were considered as valid and included for further analysis (see S1 Dataset). The remaining 195 datasets were discarded for the following reasons: 24 respondents indicated that they did not meet all of the participation criteria or did not provide informed consent. 39 datasets originated from IP addresses outside of the United States. Ten datasets originated from non-unique IP addresses. 65 respondents completed less than 90% of the questions. 37 respondents took less than 5 minutes to complete the survey, which we deemed the minimum period to be able to meaningfully complete it. 13 respondents indicated they used personal sound amplification products (PSAP) rather than HAs. Two respondents of the HA-owner survey did not specify any brand of HA or PSAP. Two HA owners did not specify how often they wore their hearing aids when watching TV/video, which was an important criterion for the TV/media listening questionnaire. Finally, three respondents indicated they had no hearing impairment in either ear.

### Statistical data analysis

Pearson’s Chi-squared test was used to test for significance of effects in contingency tables of the survey responses. The *p*-values were computed by Monte Carlo simulation [42] and, where applicable, corrected for multiple testing yielding the adjusted *p*-value, *p*_*BY*._ [43]. Analyses of covariance (ANCOVA) were performed on the responses for TV item 1 using a generalized linear mixed-effects model (GLMM) with negative binomial distribution and model comparisons via likelihood ratio tests [44]. In the modeling of responses to TV items 19 and 23, GLMMs with binomial distributions and logit link functions were used instead [44]. Least-square means were calculated to estimate factor effects in post-hoc testing [45]. Effect sizes are given in terms of log-odds differences (LODs). Log odds can be transformed to probability *P* as follows: *P* = exp(*LO*)/[1+exp(*LO*)], where *LO* stands for log odds. The coarsened exact matching method [46] used to create matched samples is described in detail in the next section.

## Respondents

### Sample characteristics

260 of the 515 respondents were HA owners, while the remaining 255 respondents did not own HAs (see S1 Table for demographic and audiologic information). Both non-HA owners and HA owners reported predominantly bilateral hearing impairments. While the prevalence of bilateral losses among HA owners (79%) was comparable to the 87% reported by Kochkin in a recent consumer satisfaction study [47], the prevalence among the non-HA owners (86%) was higher than the 61% reported in that study, which may partly be attributable to its inclusion of younger adults. The self-reported degrees of HI as well as total HHIA scores indicated stronger hearing disabilities among the HA owners than the non-HA owners (degree of HI: Pearson’s Chi-squared test, p < 0.0001; HHIA score: Kolmogorov-Smirnov test, *p* < 0.0001). For both groups, the self-reported degrees of HI were similar to those reported by Kochkin [47] and Abrams [48].

The HA owners indicated medium satisfaction with their hearing aids on the SADL scale (mean score of 4.4, SD 0.9), which was actually higher than the satisfaction that the non-HA owners expected for HA ownership on the ECHO scale (mean score of 3.7, SD 0.9; Kolmogorov-Smirnov test, *p* < 0.0001]. The HA owners’ mean SADL rating of 4.4 was slightly lower than the mean ratings of 4.7 to 5 reported in previous studies [40,41,49]. A similar trend toward lower satisfaction ratings in an online sample as compared to a clinical and lab sample has been reported previously [37]. It is possible that the SADL ratings in the present study were, to some extent, influenced by the preceding TV/media listening and HHIA questionnaires. These questionnaires might have heightened the participants’ awareness of their problems and handicaps, thereby lowering satisfaction ratings with HAs.

8% of the HA owners reported wearing their HAs “never” or “rarely” when watching TV. These 20 participants were 3.5 times more likely to indicate use of headphones or TV ears than the remaining 240 participants who reported wearing their HAs “sometimes,” “often,” or “always” when watching TV (see S2 Appendix for details). Given the high percentage of headphone or TV ear usage among these 20 participants and their infrequent use of HAs when watching TV, we believe that their responses were unlikely to reflect TV listening experiences when aided with HAs. We therefore limit the following comparative analyses to the 255 non-HA owners and 240 *HA users*, i.e., the HA owners who used their HAs at least sometimes when watching TV.

### Matched sample of non-HA owners and HA users

As described in the previous section, the HA owners had higher self-reported degrees of HI and higher unaided total HHIA scores than the non-HA owners. Furthermore, there were imbalances in gender ratios and percentages of bilateral HI between the groups (S1 Table). Thus, these potentially confounding variables could have introduced biases and affected the comparative analyses between non-HA owners and HA users such that the results might not reflect effects of HA usage on TV listening. We therefore decided to eliminate the imbalances by using coarsened exact matching (CEM), a matching method that is used in causal inference on observational data [50,51,52]. When paired with models such as GLMMs, CEM reduces model dependency. Furthermore, inferences are “double robust” in that if either the matching or analysis model is correctly specified, but not necessarily both, effect estimates are still accurate [50]. The CEM matched each of the 240 HA users to one non-HA owner as similar as possible with respect to the explanatory covariates age, gender, bilateral HI, self-reported degree of HI, and total HHIA score. Two and four coarsened histogram bins were used for participant ages and HHIA scores, respectively. 113 non-HA owners and 98 HA users, who had no close matches, were pruned, resulting in a matched sample of 284 non-HA owners and HA users. No other variables, in particular no TV related outcome variables, were consulted during the matching process to prevent inducing bias [50].

Table 1 lists the demographic and audiologic characteristics of the matched groups. These groups were identically distributed in terms of gender, bilateral HI, and degree of HI, while their age distributions as well as HHIA score distributions did not differ significantly (two-sample Kolmogorov-Smirnov tests, for age: *p* = 0.89; for HHIA score: *p* = 0.79).

**Table 1:** Demographic and audiologic characteristics of the 284 matched non-HA owners and HA users (HA owners who used their HAs at least sometimes when watching TV).

Variable	Non-HA owners (*N* = 142)		HA users (*N* = 142)	
**Age group**		(years)		(years)
	Minimum	50	Minimum	50
	25^th^ percentile	52	25^th^ percentile	52
	Median	55	Median	55
	75^th^ percentile	60	75^th^ percentile	59
	90^th^ percentile	64	90^th^ percentile	64
	Maximum	77	Maximum	79
	NR	2.8%	NR	3.5%
**Gender**		(%)		(%)
	Female	45.1	Female	45.1
	Male	54.9	Male	54.9
**Hearing impairment**		(%)		(%)
	Bilateral	85.9	Bilateral	85.9
	Unilateral	14.1	Unilateral	14.1
**Self-reported degree of hearing impairment**†		(%)		(%)
	Mild	21.8	Mild	21.8
	Moderate	63.4	Moderate	63.4
	Severe	14.8	Severe	14.8
**Mean HHIA scores**††		(score)		(score)
	Total	47.2	Total	47.7
	Social	22.5	Social	23.1
	Emotional	24.7	Emotional	24.6
**Mean global ECHO/SADL scores**		(score)		(score)
	ECHO	3.8	SADL	4.4
**Importance of hearing well when watching TV**		(%)		(%)
	Not at all important	0.7	Not at all important	0
	Slightly important	2.1	Slightly important	1.4
	Moderately important	15.5	Moderately important	12.0
	Very important	48.6	Very important	43.0
	Extremely important	32.4	Extremely important	43.7
	NR	0.7	NR	0
**HA use**				(%)
			Bilateral	60.6
			Unilateral	39.4
**Lifetime HA experience**				(%)
			Less than 6 weeks	5.6
			6 weeks to 11 months	25.4
			1 to 10 years	61.3
			More than 10 years	7.7
**Daily HA usage**				(%)
			None	0
			Less than 1 hour/day	7.7
			1 to 4 hours/day	20.4
			4 to 8 hours/day	34.5
			8 to 16 hours/day	37.3
**HA usage when watching TV**				(%)
			Sometimes	28.2
			Often	31.7
			Always	40.1
**Satisfaction with HAs when watching TV**				(%)
			Not at all satisfied	2.1
			Slightly satisfied	12.0
			Moderately satisfied	40.8
			Very satisfied	36.6
			Extremely satisfied	8.5

Results for the TV/media-related items TV12, TV21, and TV22 are also shown.

NR = no response.

^†^The rating for the worse ear was used.

^††^HHIA maximum scores for total, social, and emotional were 100, 48, and 52, respectively.

61% of the HA users reported wearing HAs on both ears, slightly fewer than the 74% in Kochkin’s [47] and Abrams’s [48] studies. The HA users seemed to be considerably experienced with HAs: 69% of HA users reported more than 11 months of HA experience while only 6% reported less than six weeks of experience. Furthermore, 72% used HAs more than 4 hours per day, while 8% used them less than 1 hour per day.

Only the results of the matched sample were included in the comparative analyses and graphs presented in the following.

## Media listening durations and viewing habits

### Results

**Fig 1:**
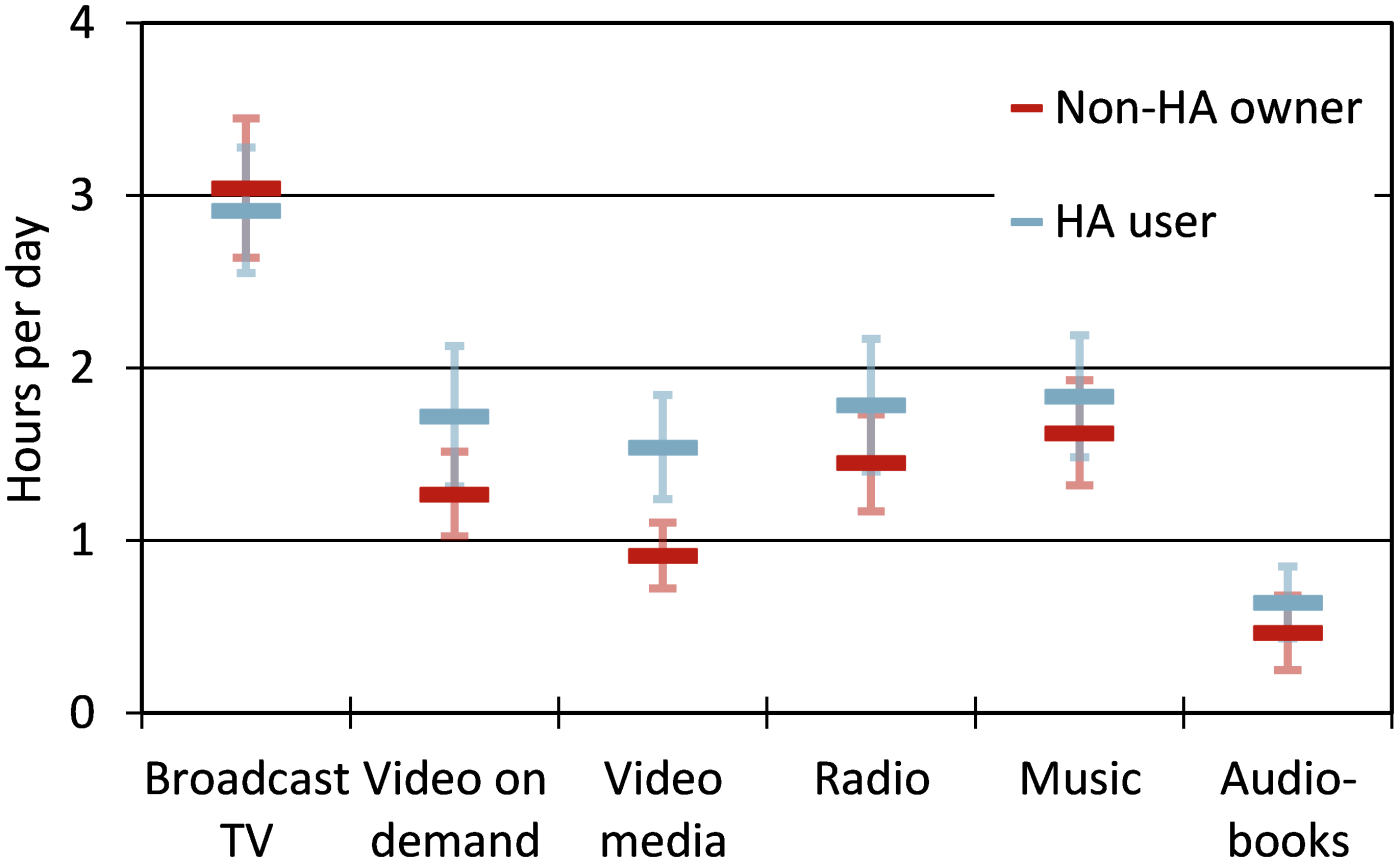
Average media consumption per day (item TV1) for the matched non-HA owners and HA users. The error bars represent 95% confidence intervals for the means.

Fig 1 shows the average responses to TV item 1, “How many hours per day (on average) do you watch or listen to the following media?” for the matched participant groups. On average, non-HA owners and HA users stated they watched TV or video 5 hours 42 min per day and listened to radio 1 hour 37 min per day (entries from five participants, who entered 20 or more hours for a single medium, were excluded). There was a significant main effect of participant group [in ANCOVA on GLMM using a negative binomial distribution, *χ*^*2*^(1) = 5.56, *p* = 0.02], while the interaction between media type and group was not significant [*χ*^*2*^(5) = 9.58, *p* = 0.09]. HA users reported that they watched TV or video for an average of 6 hours 10 min per day, 57 minutes longer than the duration reported by non-HA owners. Furthermore, there was a significant interaction between media type and age [*χ*^*2*^(6) = 15.2, *p* = 0.02]. Older participants spent more time watching broadcast TV than younger participants.

84% of the non-HA owners and HA users considered hearing well when watching TV as “very important” or “extremely important” (item TV12, see Table 1, no significant group difference, *p* = 0.3). However, 55% of the HA users were “not at all satisfied,” “slightly satisfied,” or “moderately satisfied” with their HAs when watching TV (item TV22, see Table 1).

The non-HA owners and HA users answered the questions about their media listening environments and viewing equipment very similarly (see S3 Appendix for details). They watched TV mostly at home in the living room and sometimes in the bedroom, with an average room size of about 20 m^2^. The majority (79%) used the loudspeakers in the TV. Regarding the types of shows watched, male participants watched sports significantly more frequently than female participants, without significant differences between non-HA owners and HA users (S3 Appendix).

### Discussion

The media listening durations indicated by our participants are consistent with Nielsen [53], who reported that adults of ages 50 to 64 years in the United States in 2015 spent on average 6 hours 29 min per day watching TV or video and 2 hours 8 min per day listening to radio. Similar to our results, Nielsen also observed that older adults spent more time watching live TV than younger adults.

## Problems encountered when watching TV

### Results

**Fig 2:**
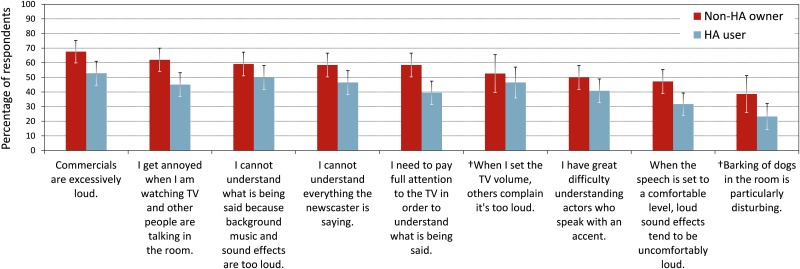
Problems encountered when watching TV (item TV23) in terms of percentages of respondents. “†” marks a response option that was added later and thus is based on a smaller dataset. The error bars represent 95% confidence intervals for the means.

**Fig 3:**
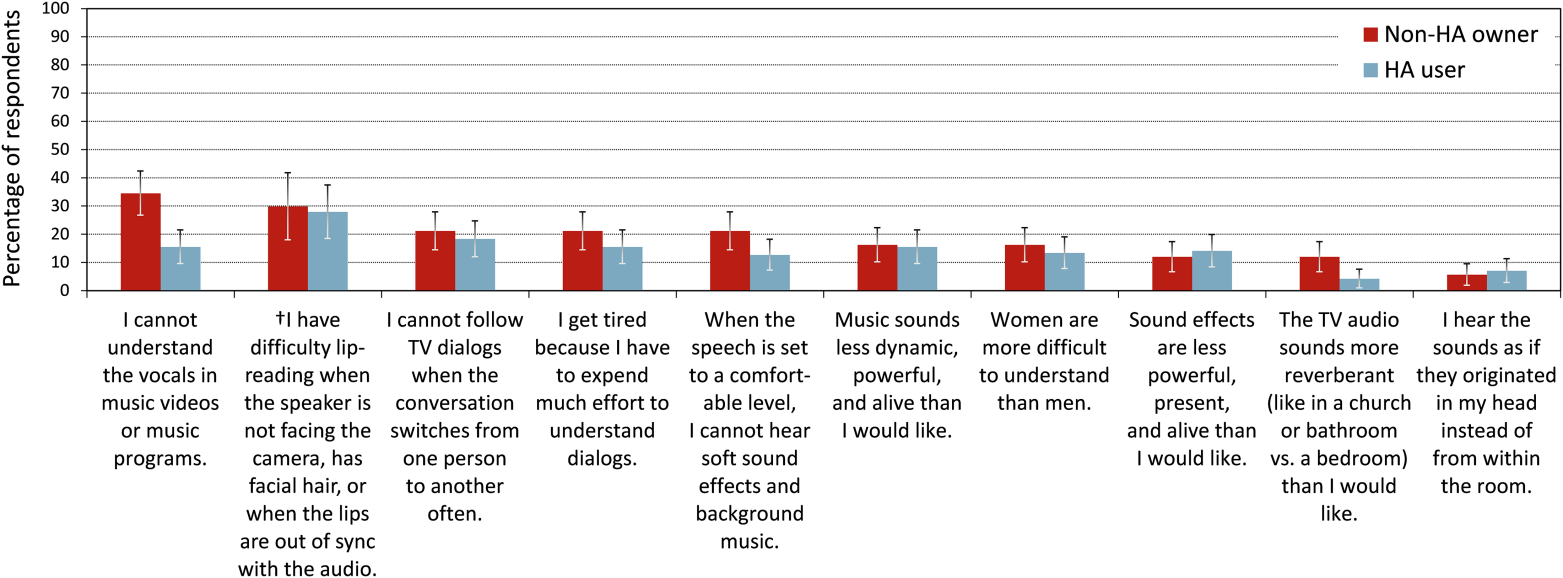
Continued from Fig 2, problems encountered when watching TV (item TV23) in terms of percentage of respondents. “†” marks response options that were added later and thus are based on smaller datasets. The error bars represent 95% confidence intervals for the means.

TV item 23 inquired about problems that the participants experienced when watching TV. Figs 2 and 3 depict, for each of the 19 closed-set problems, the percentages of participants who encountered that problem (in descending order). Seven of the 19 problems were encountered by more than 45% of the participants (Fig 2). To explore the significance of demographic and audiologic factors, the data were subjected to ANCOVA on GLMMs with binomial distributions in backward elimination. The main effects of TV problem [*χ*^*2*^(18) = 904, *p* < 0.0001] and participant group were significant [*χ*^*2*^(1) = 18.0, *p* < 0.0001]. Overall, non-HA owners were more likely to indicate problems when watching TV than HA users (LOD of 0.57). Furthermore, participants with bilateral HI were more likely to indicate problems than those with unilateral impairments [*χ*^*2*^(1) = 10.2, *p* = 0.001, LOD of 0.62], and problem probability increased with greater degree of HI [*χ*^*2*^(2) = 18.0, *p* < 0.001, LODs of 0.47 for moderate vs. mild losses and 0.96 for severe vs. mild losses]. There was a significant interaction between degree of HI and participant group [*χ*^*2*^(2) = 6.46, *p =* 0.04]: Non-HA owners were more likely to indicate problems when watching TV than HA users if they had mild or moderate impairments (LODs of 0.74 and 0.69, respectively), but there was no significant group difference for participants with severe hearing impairments (LOD of -0.22). Age had no significant influence (*p* = 0.83).

Although the interaction of participant group with TV problem was not significant [*χ*^*2*^(18) = 24.7, *p* = 0.13], it was retained for post-hoc tests on the group differences. The following differences were significant at a 95% confidence level without multiple testing correction. More non-HA owners than HA users reported that commercials were excessively loud (LOD 0.75), reported annoyance when they were watching TV and other people were talking in the room (LOD 0.83), reported difficulties understanding everything the newscaster was saying (LOD 0.59), indicated they needed to pay full attention to the TV in order to understand what was being said (LOD 0.93), reported discomfort with loud sound effects (LOD 0.80) or inaudibility of soft sound effects and background music (LOD 0.72) when the speech was set to a comfortable level, reported difficulty understanding the vocals in music videos or music programs (LOD 1.26), and indicated that the audio sounded more reverberant than they would have liked (LOD 1.27; however, this problem was only indicated by 8% of the participants).

Furthermore, gender was a significant factor [*χ*^*2*^(1) = 9.76, *p* = 0.002]. Overall, more female than male participants reported problems (LOD of 0.41). The interaction of gender with TV problem was not significant [*χ*^*2*^ (18) = 27.0, p = 0.08].

The HHIA score was a significant predictor of TV problems, with the probability of TV problems increasing with increasing HHIA score [*χ*^*2*^(1) = 16.1, *p* < 0.0001]. However, since it was conceptually very similar to TV item 23 (both inquired about the frequency of hearing problems), the HHIA score was not included as a predictor in the full model of the backward elimination procedure.

In addition to the above demographic and audiologic factors, TV specific factors were included in a larger model. The effect of listening in an environment with mostly carpeted floor (TV6) was significant [*χ*^*2*^(1) = 10.8, *p* = 0.001], including its interactions with the main effects participant group and TV problem [*χ*^*2*^(1) = 3.90, *p* < 0.05; and *χ*^*2*^(18) = 29.0, *p* < 0.05, respectively]. Participants without floor carpets (26% of all participants, S3 Appendix) were more likely to report problems than those with carpets, with the effect being stronger for non-HA owners than for HA users [LODs of 0.76 and 0.19, respectively]. With regard to each TV problem, the largest effects of carpet were fewer reports of getting annoyed when other people were talking in the room, others complaining the TV was set too loud, loud sound effects being too loud, understanding vocals in music, being disturbed by dogs barking, getting tired because of effortful listening, and TV sounding too reverberant (most significant effect). Furthermore, the frequency of using the remote control to change TV volume (TV13) was a significant factor [*χ*^*2*^(4) = 15.9, *p =* 0.003], including its interaction with the main effect TV problem [*χ*^*2*^(72) = 119, *p* < 0.001]. The more often participants used the remote control, the higher was the probability of them reporting problems when watching TV [LOD of 1.66 between using the remote control “never” and “every few minutes”]. Put differently, the more participants experienced problems when watching TV, the more often they used their remote controls for assistance. The largest effects were observed for the following TV problems: commercials being excessively loud, background music and sound effects being too loud, others complaining the TV was set too loud, and loud sound effects being too loud. The frequency of remote control usage was associated with the degree of self-reported HI [Pearson’s Chi-squared test, *p* = 0.002], i.e., the stronger the participants’ hearing impairments, the more frequently they made use of their remote controls. Finally, the following TV specific factors were not significant (*p* > 0.05): duration spent watching TV/video (TV1), room size (TV5), viewing distance (TV7), type of speakers (TV8), type of external speakers (TV9), background noise in listening environment (TV10), and frequency of using CC (TV20).

In contrast to the above analyses on the groups matched on demographic and audiologic characteristics, the significance of HA related factors was assessed using the data from the HA owners. To increase statistical power, the full dataset of 260 HA owners was used here. The HA related factors were tested on a GLMM that included the same basic demographic and audiologic factors that were found significant above (gender, bilateral HI, degree of HI, and TV problem). The global SADL score was a significant predictor [*χ*^*2*^(1) = 11.7, *p* < 0.001]: the probability of TV problems decreased with increasing SADL scores. In addition, the daily HA use duration (additional SADL item) was a significant factor [*χ*^*2*^(4) = 19.1, *p* < 0.001], with the probability of TV problems increasing with greater HA use duration [LOD of 0.72 between HA use of “1 to 4 hours per day” and “8 to 16 hours per day”]. Put differently, the more participants experienced problems when watching TV, the lower were their SADL scores and the higher were their HA use durations. The frequency of using HAs when watching TV (TV21) and the satisfaction with HAs when watching TV (TV22) were not significant (*p* = 0.2 and *p* = 0.4, respectively).

A few other problems were mentioned in response to the open-ended question TV24. The two problems that were mentioned most frequently were outside noise interference and a variety of problems with CC such as poor transcription, high display rate, poor visibility, etc.

**Fig 4:**
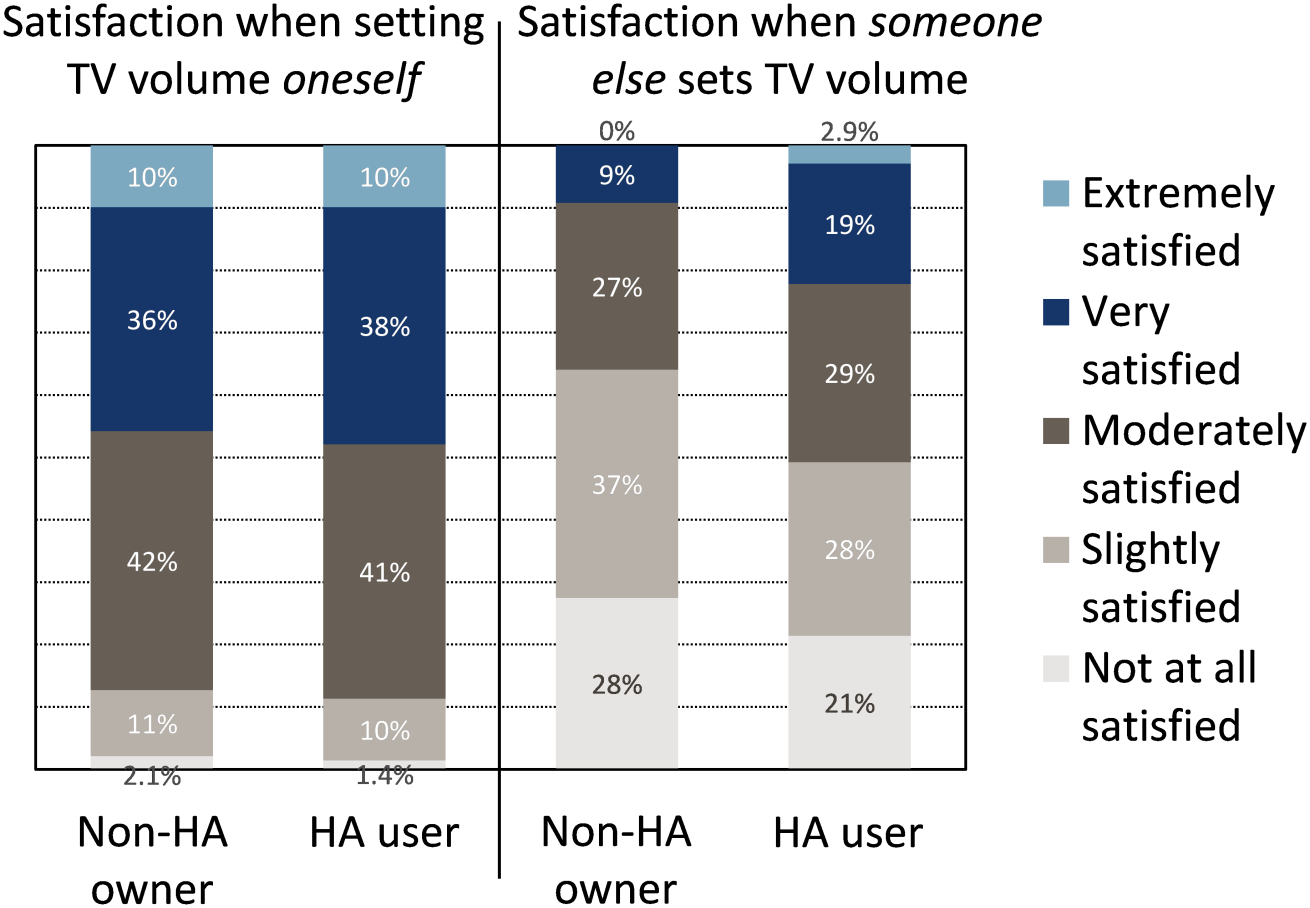
Satisfaction with TV loudness. Satisfaction when participants set the TV volume themselves (left), and when someone else with good hearing set the volume (right), items TV15 and TV16, respectively.

Three of the most frequently reported problems in response to item TV23 were related to TV loudness. Two other questionnaire items queried the participants explicitly about their satisfaction with TV loudness, one when they set the TV volume themselves (TV15), and another when someone else with good hearing set the volume (TV16) (see Fig 4). Both non-HA owners and HA users were significantly more satisfied with loudness when they set it themselves than if it was set by someone else with good hearing (*p*_*BY*_ < 0.0001). In the latter situation, HA users were significantly more satisfied than non-HA owners (*p*_*BY*_ < 0.05).

**Fig 5:**
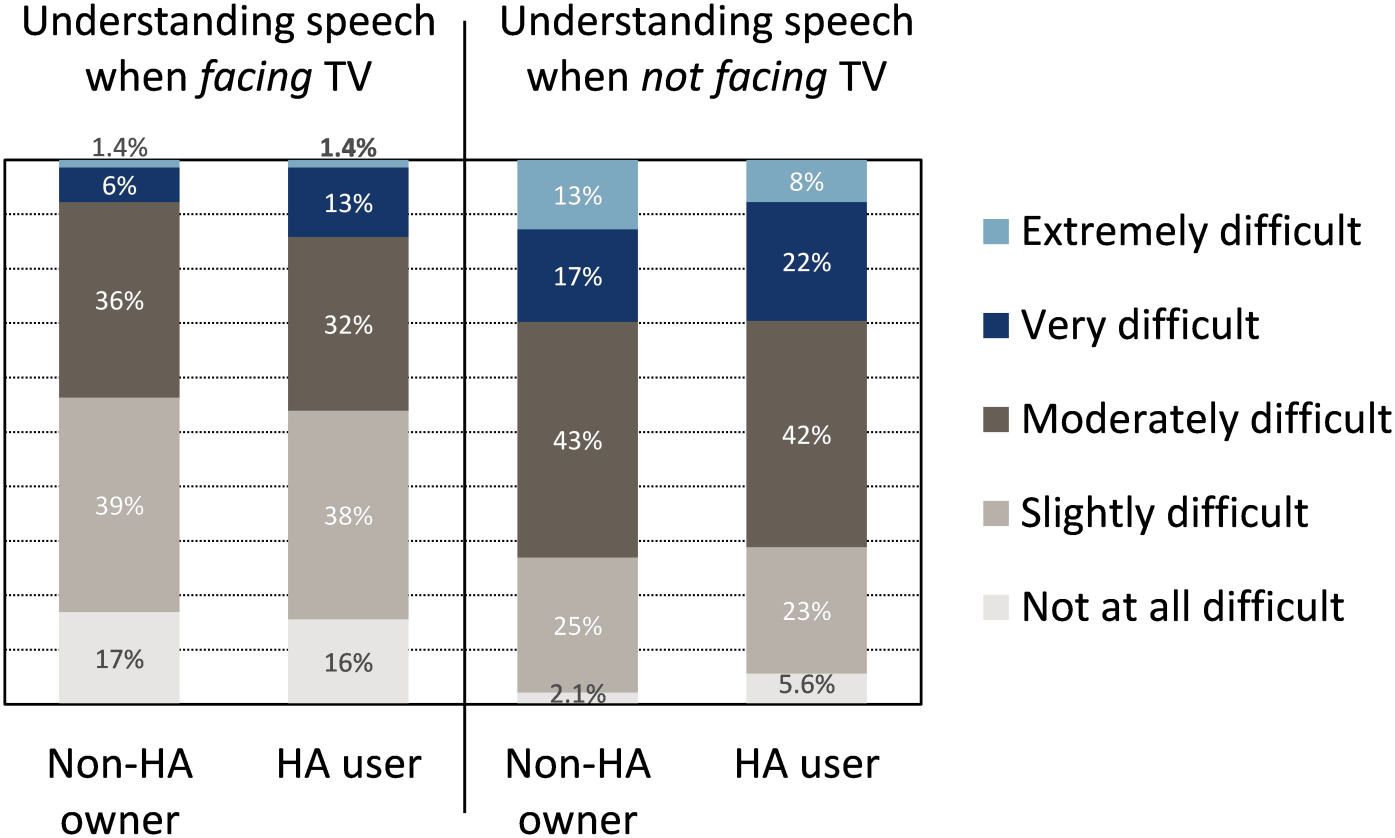
Difficulty understanding speech on TV. Difficulty when participants were facing the TV (left), and when they were not facing the TV (right), items TV17 and TV18, respectively.

Fig 5 shows the responses to items TV17 and TV18, which asked the participants to rate the difficulty of understanding speech on TV, when they were facing the TV (TV17), and when they were not facing the TV (TV18). Both non-HA owners and HA users rated difficulty significantly greater when not facing the TV than when facing the TV (*p*_*BY*_ < 0.0001). There were no significant group differences (*p*_*BY*_ > 0.7).

### Discussion

HA users indicated fewer difficulties when watching TV than non-HA owners, even after controlling for covariates by matching the samples and including covariates as predictors in analysis models. This suggests that HA usage alleviated difficulties with TV listening. HA users experienced fewer problems than non-HA owners with understanding newscasters, which may be attributable to HA amplification enhancing audibility of speech. Furthermore, fewer of the HA users than non-HA owners reported needing to pay full attention to understand speech and getting annoyed when other people were talking in the room. This is consistent with findings that HA amplification and sound processing can free cognitive resources and reduce listening effort [54]. Apart from reduced listening effort, not needing to pay full attention could also be due to HA users potentially being less reliant on visual cues such as lip reading cues. HA dynamic range compression may explain why fewer HA users than non-HA owners perceived loud sounds effects and commercials as too loud and soft sounds as inaudible. A large group difference was observed with regard to more non-HA owners than HA users not being able to understand vocals in music. This is a surprising result, the reasons for which remain unclear.

It is important to note that the exclusion of the 20 HA owners who did not use their HAs when watching TV was conservative with regard to the observed group differences between non-HA owners and HA users, as the 20 excluded HA owners indicated even fewer problems than the other HA owners (S2 Appendix). One might argue that the matching of non-HA owners and HA users in terms of their HHIA scores and self-reported degree of HI was not sufficient to equalize the groups in terms of their unaided hearing impairments, as the non-HA owners might have underestimated their handicaps, given their decision not to obtain HAs, and the HA users might have had difficulty rating their handicaps without HAs. However, this explanation is unlikely to account for the observed differences in perceived difficulties, as an analysis of the full groups of 255 non-HA owners and 260 HA users yielded the same outcome: HA users experienced significantly fewer difficulties than the non-HA owners [*χ*^*2*^(1) = 14.8, *p* < 0.001] despite the HA users reporting more severe handicaps than the non-HA owners by an average 16-point difference on the HHIA.

It is important to note that, although HA users reported significantly fewer difficulties than non-HA owners when watching TV, the seven most frequently indicated problems were still encountered by more than 39% of the HA users. Furthermore, 49% of the HA users were only slightly or not at all satisfied with the loudness of the TV when a NH person set the TV volume, and 30% of them found it very difficult or extremely difficult to understand speech when not facing the TV. Understanding speech was rated easier when facing the TV, presumably due to the availability of lip reading cues, visual context, and CC when facing the screen.

Women were more likely to report problems than men, which is consistent with previous studies on TV listening [20,55]. One possible reason for women indicating problems more frequently than men could have been different viewing preferences (if women watched the more challenging materials more often than men). However, the only significant gender difference in terms of viewing preferences was women watching sports broadcasts less frequently than men. Furthermore, women also indicated problems with understanding newscasts significantly more frequently than men, which is considered the least problematic of all program types [19,20]. Thus, viewing preferences are unlikely to account for the observed gender differences in the present study.

In addition to gender effects, problem probability increased with greater degree of self-reported HI and was larger for participants with bilateral than unilateral impairments. Participant age had no significant influence. It should be noted that the study sample was obtained by convenience sampling and thus may not have been completely representative of the HI population at large, at least with regard to the participants’ ages (90% of the participants were between 50 and 64 years old). On the other hand, Williger and Hannemann’s [20] study sample included older participants than the present study, and they did not observe a significant age effect on difficulties with TV listening either. Moreover, with regard to self-reported degrees of HI, media listening durations, and viewing equipment, the present study sample was consistent with past research [47,48,53,56]. It is worth noting that a power analysis based on bootstrap sampling from the matched participant groups showed that 190 study participants would be needed to discover the significant group effect with a probability of 0.9 (the required sample sizes for powers of 0.5 and 0.8 would be 70 and 130 participants, respectively).

The TV problem most frequently encountered by both non-HA owners and HA users was commercials being excessively loud, and the most frequently mentioned reason for using the remote control to change volume was to decrease commercials, a pattern consistent with past research [55]. Complaints about loudness of TV commercials have led to loudness legislations and recommendations across the globe since 2010, requiring commercials to have the same average loudness as the accompanying programs [57]. Declining viewer complaints suggest that these steps have been at least partly effective in curbing the loudness of TV commercials [22, 58,59]. This interpretation is also consistent with declining online search interest since 2010 for terms such as “loud commercials,” “commercials too loud,” and “why are commercials so loud” [60]. Nevertheless, 60% of the participants in the present study indicated commercials were excessively loud.

A number of participants pointed out in their open responses that they found the barking of dogs particularly disturbing when watching TV. Therefore, we decided to add this as a response option. Indeed, 29% of the respondents indicated they experienced this problem, which is a surprisingly high percentage given that only 37% to 44% of U.S. households own a dog [61,62]. It remains unknown whether HI viewers are particularly susceptible or whether a similar percentage of NH viewers would be disturbed by barking.

We suggested in the introduction that room acoustic effects (e.g., reverberation, suboptimal placement of TV loudspeakers, etc.) could aggravate speech perception problems of HI viewers. The observed effect of floor carpeting is consistent with this hypothesis: Participants with carpeted floor in the TV viewing room were less likely to experience problems than those without carpets. Carpet can provide significant sound absorption at mid and high frequencies. It thereby lessens room acoustic contributions such as comb-filtering and coloration due to floor reflections, reverberation in general, and it can also dampen intrusive background noises [63]. Thus, carpets may indeed have decreased participants’ problems when watching TV. On the other hand, there were no significant effects of room size, viewing distance, or type of speakers used. It is possible that acoustic effects of these latter factors were obscured by other influences. For example, the probability of TV problems as a function of viewing distance as well as room size showed ∨-shaped trends, with problem frequencies largest for the smallest and largest viewing distances and room sizes, respectively. 16% of the participants in the present study indicated that they moved closer to the TV when experiencing difficulties with understanding speech. Thus, it is possible that those who experienced the largest difficulties tended to move closest to the TV. This is similar to the frequency of remote control usage. While remote control usage can improve the TV listening experience, the observed effects were actually opposite in the present study, which is consistent with the interpretation that those who experienced the largest difficulties were also the ones who used their remote controls most often. Furthermore, viewing distance, room size, and type of speakers may have co-varied with other possibly relevant sociodemographic factors such as income, household size, educational attainment, etc. In contrast, floor carpeting may have co-varied less with such unobserved nuisance variables. Interestingly, Williger and Hannemann [20] observed an effect of TV placement: Participants with wall-mounted TVs reported fewer difficulties than participants with freestanding TVs or TVs placed in wall units. This observation and the effect of carpet in the present study suggest that the acoustics of the viewing environment may affect the TV listening experiences of HI viewers.

## Compensation strategies

### Results

**Fig 6:**
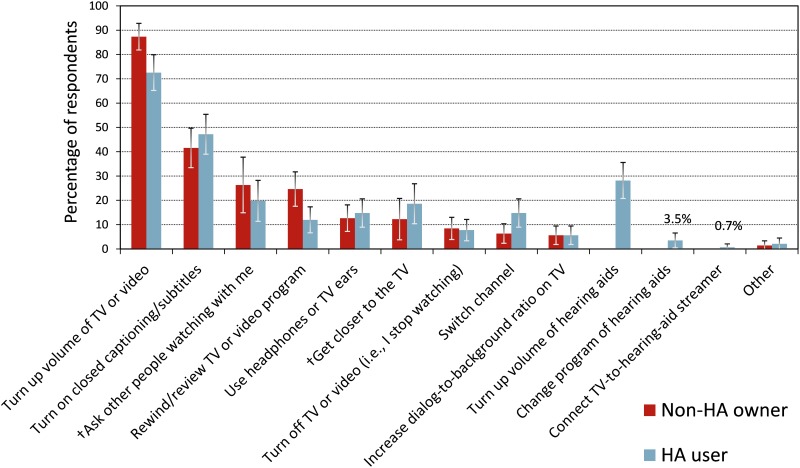
Compensation strategies. Strategies employed by the participants when they experienced difficulties with understanding speech on TV (item TV19). “†” marks response options that were added later and thus are based on smaller datasets. The error bars represent 95% confidence intervals for the means.

TV item 19 inquired about compensation strategies that the participants used when they experienced problems with understanding speech on TV. Fig 6 shows the percentages of participants who employed the given strategies (in descending order). The most frequently used compensation strategy was turning up the volume of the TV, followed by switching on CC. To explore the significance of demographic and audiologic factors, ANCOVA were performed on GLMMs with binomial distributions in backward elimination. The main effect of participant group was not significant [*χ*^*2*^(1) = 1.04, *p* = 0.3]. However, there was a significant interaction between compensation strategy and participant group [*χ*^*2*^(9) = 27.2, *p* < 0.01]. Non-HA owners were more likely to turn up the TV volume than HA users [87% vs. 73%, LOD of 1.01], were more likely to rewind or review the TV program [LOD 0.94], and were less likely to switch channels [LOD 0.97] (post-hoc tests significant at 95% confidence level without multiple testing correction). 28% of the HA users turned up the HA volume, with 5% turning up only the HA volume and not the TV volume. 23% of the HA users turned up neither TV nor HA volume, which is a larger percentage than the 13% of non-HA owners who did not turn up the TV volume. Thus, the difference between the non-HA owners and HA users in terms of turning up the TV volume can be attributed to some HA users turning up the HA volume instead of the TV volume and a larger percentage of HA users than non-HA owners not turning up any volume.

The self-reported degree of HI was a significant factor [*χ*^*2*^(2) = 9.85, *p* < 0.01]. Participants with moderate and severe losses were more likely to apply compensation strategies than those with mild losses. Age, gender, and whether the hearing impairment was bilateral or unilateral had no significant influences on compensation strategies (*p* > 0.05).

The frequency of external background noise in the TV viewing environment (TV10) was also a significant factor [*χ*^*2*^(4) = 14.1, *p* < 0.01]. Participants who experienced background noise more frequently, were more likely to apply compensation strategies.

Only 3.5% of the HA users indicated that they changed the HA program when experiencing difficulties with understanding speech. The response option “Other” was selected by 2% of the participants. The “other” strategies included “reading lips,” “closing doors,” “telling everyone to shut up,” and behaviors consistent with resignation such as “muting the TV” or “living with it”.

44% of the participants indicated that they turned on CC when experiencing difficulty with understanding speech. Item TV20 asked a closely related question, inquiring how often the participants used CC when watching TV. The reported CC usage frequencies were: “never” (18%), “rarely” (22%), “sometimes” (33%), “often” (16%), and “always” (11%), with no significant group differences (*p* = 0.19). The percentage of the participants who used CC at least sometimes (60%) was larger than the percentage of participants who indicated they turned on CC when they had difficulties understanding speech (44%) in response to the preceding item TV19. It is possible that those who used CC all the time did not select ‘turning on CC’ as a response to item TV19 simply because they used CC all the time.

As mentioned above, the most frequently employed compensation strategy was turning up the TV volume. This is related to item TV13, which asked participants how often they typically used the remote control to change the TV volume. The reported usage frequencies were: “never” (6%), “only once after switching on the device” (15%), “at the beginning of a new program” (31%), “before/after commercials” (29%), and “every few minutes” (20%), with no significant group differences (*p* = 0.39). The following open-ended item TV14 inquired about the reasons for changing the volume. In order of descending frequency, the stated reasons were to decrease (or mute) commercials, to increase soft sections, to adjust the volume for loudness differences between programs, channels, or media, to decrease sections that were too loud (sound effects or background music), and to adjust for external noise such as noises from the street, AC, space heaters, cooking, dogs barking, children playing, or people talking.

### Discussion

When asked about strategies that they employed when experiencing difficulties with understanding speech, fewer HA users than non-HA owners indicated they turned up the TV volume or rewound the TV program. However, HA users were more likely to switch channels than non-HA owners. 44% of the participants in our study indicated they switched on CC when experiencing difficulties. These are positive news, considering that CC is effective in improving speech understanding [13]. Remarkably, there was low reported usage among the HA users of audio streaming accessories such as TV-to-HA streamers (6%, see S3 Appendix), technologies that can provide high-quality TV audio signals with adequate gain, spatial (stereo) cues, and without the interference of room acoustics. However, with increased focus on connectivity and accessibility among HA manufacturers and users [64], direct audio streaming may become commonplace in the future.

## Conclusions

In an online survey on TV listening, HA users reported that they watched TV or video on average for 6 hours 10 min per day, 57 minutes longer than the duration reported by non-HA owners. Furthermore, HA users indicated fewer difficulties when watching TV than non-HA owners, even after controlling for covariates. This indicates that HA usage alleviated difficulties with TV listening. Nevertheless, the seven most frequently reported problems were still encountered by more than 39% of the HA users. Difficulties increased with greater self-reported unaided hearing disability. Female participants indicated more problems than male participants, and those with carpeted floors reported fewer difficulties than those without carpets. The most frequently used compensation strategies were changing TV or HA volumes and using closed captioning. Only few HA users used audio streaming accessories. The present study has raised new questions that could not be answered given the exploratory nature of the investigation. For example, does floor carpeting reduce difficulties with TV listening or was the observed association due to a common, unobserved cause? Why did HA users report less difficulty with understanding vocals in music than non-HA owners? Why were women more likely to report difficulties than men? Further studies are needed to answer these questions and to inform interventions or recommendations that will improve the TV listening experiences of unaided and aided HI viewers.

## Supporting information

S1 Appendix TV/media listening questionnaire. (PDF)

S2 Appendix Respondents who wear their hearing aids rarely or never when watching TV. (PDF)

S3 Appendix Media listening environment, viewing equipment, and types of shows watched. (PDF)

S1 Dataset Survey data. (XLSX)

S1 Table Demographic and audiologic characteristics of the 515 respondents. (PDF)
