# Supplementary material for: TV listening and hearing aids
Source: PLoS One. 2018 Jun 29;13(6):e0200083. doi: 10.1371/journal.pone.0200083 (PMC6025866; doi:10.1371/journal.pone.0200083)
Supplement: S1 Appendix — (PDF) [file pone.0200083.s001.pdf]

## S1 Appendix – TV/media listening questionnaire

In the following, the "*"-symbol marks items or response options that were only present in the survey for the HA owners. These items and options were absent in the survey for the non-HA owners. "†" marks response options that were added after the first batch of respondents had completed the surveys. "○" stands for an exclusive option and "☐" stands for multiple options ("select all that apply").

## TV/media listening questionnaire

Instructions: Listed below are questions relating to your experiences when listening to electronic media (e.g., TV, movies, online videos, radio). Please select the item which best matches your response to each question.

TV1) How many hours per day (on average) do you watch or listen to the following media?

| | |
|---|---|
| Broadcast TV | ____ |
| Video on demand (online video services, pay-per view, etc.) | ____ |
| Video media (DVDs, Blu-rays, video file formats such as .avi, .flv., .wmv, .mov,. mp4) | ____ |
| Radio | ____ |
| Music | ____ |
| Audiobooks | ____ |

TV2) How often do you watch TV or video in the following locations?

| | Never | Rarely | Sometimes | Often | Always |
|---|---|---|---|---|---|
| At home | ○ | ○ | ○ | ○ | ○ |
| At work | ○ | ○ | ○ | ○ | ○ |
| On public transport (e.g., bus, train) | ○ | ○ | ○ | ○ | ○ |
| In the car | ○ | ○ | ○ | ○ | ○ |

TV3) How often do you watch TV or video on the following devices?

| | Never | Rarely | Sometimes | Often | Always |
|---|---|---|---|---|---|
| TV set | ○ | ○ | ○ | ○ | ○ |
| PC/Laptop | ○ | ○ | ○ | ○ | ○ |
| Tablet computer | ○ | ○ | ○ | ○ | ○ |
| Mobile phone | ○ | ○ | ○ | ○ | ○ |

TV4) At home, how often do you watch TV in the following rooms?

| | Never | Rarely | Sometimes | Often | Always |
| --- | --- | --- | --- | --- | --- |
| Living room | O | O | O | O | O |
| Dining room | O | O | O | O | O |
| Bedroom | O | O | O | O | O |
| Kitchen | O | O | O | O | O |

TV5) How large is the room where you watch TV/video most of the time?

- O Less than 140 square feet (< 13 square meters)
- O Between 140 and 220 square feet (13 to 20 square meters)
- O Between 220 and 320 square feet (20 to 30 square meters)
- O Between 320 and 420 square feet (30 to 40 square meters)
- O More than 420 square feet (> 40 square meters)

TV6) Does the room where you watch TV/video most of the time have mostly carpeted floor?

- O Yes
- O No

TV7) What is your typical viewing distance (that is, the distance between you and the screen)?

- O Less than 5 feet (< 1.5 meters)
- O Between 5 and 8 feet (1.5 to 2.4 meters)
- O Between 8 and 12 feet (2.4 to 3.7 meters)
- O More than 12 feet (> 3.7 meters)

TV8) What type of speakers do you use? (Please select all that apply.)

- ☐ Speakers in the TV
- ☐ External loudspeakers
- ☐ Headphones or TV ears [(without hearing aids)*]
- ☐ Induction loop system*
- ☐ TV-to-hearing-aid streamer (Phonak TV link, Oticon ConnectLine TV, ReSound Unite TV streamer, Starkey Surflink Media, Widex TV-DEX, etc.)*

TV9) If you use external loudspeakers, which type do you use?

- O Sound bar (single, wide, external loudspeaker enclosure typically placed in front of the TV)
- O Two external loudspeakers (left and right)
- O Three external loudspeakers (left, center, and right)
- O Surround sound system (5 or more loudspeakers)
- O Other (please specify)

_ _ _ _ _ _ _ _ _ _ _ _ _ _ _ _ _ _ _ _

TV10) How often does the listening environment in which you watch TV or video exhibit background noise (e.g., other people talking, noise from other rooms, noise from outside)?

O Never O Rarely O Sometimes O Often O Always

TV11) How often do you watch the following shows when watching TV/video?

| | Never | Rarely | Sometimes | Often | Always |
|---|---|---|---|---|---|
| Documentaries | O | O | O | O | O |
| Drama | O | O | O | O | O |
| Game shows | O | O | O | O | O |
| News | O | O | O | O | O |
| Sitcoms | O | O | O | O | O |
| Sports | O | O | O | O | O |

TV12) How important is it for you to hear well when watching TV/video?

- O Extremely important
- O Very important
- O Moderately important
- O Slightly important
- O Not at all important

TV13) While watching a program on TV/video, how often do you typically use the remote control to change volume?

- O Never
- O Only once after switching on the device
- O At the beginning of a new program
- O Before/after commercials
- O Every few minutes

TV14) If you typically use the remote control to change the volume, please indicate why you would change the volume:

_ _ _ _ _ _ _ _ _ _ _ _ _ _ _ _ _ _ _ _

TV15) How satisfied are you typically with the loudness of the TV/video when you set the volume yourself?

- O Extremely satisfied
- O Very satisfied
- O Moderately satisfied
- O Slightly satisfied
- O Not at all satisfied

TV16) How satisfied are you typically with the loudness of the TV/video when someone else with good hearing sets the volume?

- O Extremely satisfied
- O Very satisfied
- O Moderately satisfied
- O Slightly satisfied
- O Not at all satisfied

TV17) When you are facing the TV or video, how difficult is it for you to understand speech on the TV or video?

- O Extremely difficult
- O Very difficult
- O Moderately difficult
- O Slightly difficult
- O Not at all difficult

TV18) When you are NOT facing the TV or video, how difficult is it for you to understand speech on the TV or video?

- O Extremely difficult
- O Very difficult
- O Moderately difficult
- O Slightly difficult
- O Not at all difficult

TV19) When you have difficulty understanding speech on TV/video, what do you typically do to "fix" the problem? (Please select all that apply.)

- ☐ Turn up volume of TV or video
- ☐ Turn on closed captioning/subtitles
- ☐ Increase dialog-to-background ratio on TV
- ☐ Use headphones or TV ears
- ☐ Switch channel
- ☐ Turn off TV or video (i.e., I stop watching)
- ☐ Rewind/review TV or video program
- ☐ Ask other people watching with me†
- ☐ Get closer to the TV†
- ☐ Turn up volume of hearing aids*
- ☐ Change program of hearing aids*
- ☐ Connect TV-to-hearing-aid streamer*
- ☐ Other

  _ _ _ _ _ _ _ _ _ _ _ _ _ _ _ _ _ _ _ _

TV20) How often do you use closed captioning/subtitles when watching TV/video?

- O Never
- O Rarely
- O Sometimes
- O Often
- O Always

TV21) How often do you wear your hearing aid(s) when watching TV/video?*

- O Never
- O Rarely
- O Sometimes
- O Often
- O Always

TV22) How satisfied are you with your hearing aid(s) when watching TV/video?*

- ○ Extremely satisfied
- ○ Very satisfied
- ○ Moderately satisfied
- ○ Slightly satisfied
- ○ Not at all satisfied

TV23) Which of these problems do you encounter when watching TV/video? (Please select all that apply.)

- ☐ I cannot understand everything the newscaster is saying.
- ☐ I cannot understand what is being said because background music and sound effects are too loud.
- ☐ I have great difficulty understanding actors who speak with an accent.
- ☐ Women are more difficult to understand than men.
- ☐ I cannot follow TV dialogs when the conversation switches from one person to another often.
- ☐ I need to pay full attention to the TV in order to understand what is being said.
- ☐ I get annoyed when I am watching TV and other people are talking in the room.
- ☐ I get tired because I have to expend much effort to understand dialogs.
- ☐ Barking of dogs in the room is particularly disturbing.†
- ☐ I have difficulty lip-reading when the speaker is not facing the camera, has facial hair, or when the lips are out of sync with the audio.†
- ☐ I cannot understand the vocals in music videos or music programs.
- ☐ Sound effects are less powerful, present, and alive than I would like.
- ☐ Music sounds less dynamic, powerful, and alive than I would like.
- ☐ When the speech is set to a comfortable level, loud sound effects tend to be uncomfortably loud.
- ☐ When the speech is set to a comfortable level, I cannot hear soft sound effects and background music.
- ☐ Commercials are excessively loud.
- ☐ When I set the TV volume, others complain it’s too loud.†
- ☐ I hear the sounds as if they originated in my head instead of from within the room.
- ☐ The TV audio sounds more reverberant (like in a church or bathroom vs. a bedroom) than I would like.

TV24) What other problems do you encounter when watching TV/video? You may describe up to 4 problems. Please rate the frequency of the problem by including one of the following numbers at the beginning of your response (1=never, 2=rarely, 3=sometimes, 4=often, 5=always).

Problem #1 _ _ _ _ _ _ _ _ _ _ _ _ _ _ _ _ _ _ _ _
Problem #2 _ _ _ _ _ _ _ _ _ _ _ _ _ _ _ _ _ _ _ _
Problem #3 _ _ _ _ _ _ _ _ _ _ _ _ _ _ _ _ _ _ _ _
Problem #4 _ _ _ _ _ _ _ _ _ _ _ _ _ _ _ _ _ _ _ _

TV25) Do you remember a specific film scene, broadcast, or video clip, in which it was difficult to understand the speech? (Be specific: name the program, describe the scene and/or provide the time code.)†

_ _ _ _ _ _ _ _ _ _ _ _ _ _ _ _ _ _ _ _

TV26) How often do you listen to the radio in the following locations?

| | Never | Rarely | Sometimes | Often | Always |
|---|---|---|---|---|---|
| At home | O | O | O | O | O |
| At work | O | O | O | O | O |
| On public transport (e.g., bus, train) | O | O | O | O | O |
| In the car | O | O | O | O | O |

TV27) How often do you listen to the radio on the following devices?

| | Never | Rarely | Sometimes | Often | Always |
|---|---|---|---|---|---|
| Receiver radio | O | O | O | O | O |
| High fidelity (hi-fi)/home stereo system | O | O | O | O | O |
| PC/Laptop | O | O | O | O | O |
| Tablet computer | O | O | O | O | O |
| Mobile phone | O | O | O | O | O |

TV28) When listening to the radio, what are the most frequent problems you encounter? You may describe up to 4 problems. Please rate the frequency of the problem by including one of the following numbers at the beginning of your response (1=never, 2=rarely, 3=sometimes, 4=often, 5=always).

Problem #1 _ _ _ _ _ _ _ _ _ _ _ _ _ _ _ _ _ _ _ _ _
Problem #2 _ _ _ _ _ _ _ _ _ _ _ _ _ _ _ _ _ _ _ _ _
Problem #3 _ _ _ _ _ _ _ _ _ _ _ _ _ _ _ _ _ _ _ _ _
Problem #4 _ _ _ _ _ _ _ _ _ _ _ _ _ _ _ _ _ _ _ _ _

TV29) How often do you listen to music in the following locations?

| | Never | Rarely | Sometimes | Often | Always |
|---|---|---|---|---|---|
| At home | O | O | O | O | O |
| At work | O | O | O | O | O |
| On public transport (e.g., bus, train) | O | O | O | O | O |
| In the car | O | O | O | O | O |
| Live performance | O | O | O | O | O |

TV30) How often do you listen to music on the following devices?

| | Never | Rarely | Sometimes | Often | Always |
|---|---|---|---|---|---|
| High fidelity (hi-fi)/home stereo system | O | O | O | O | O |
| PC/Laptop | O | O | O | O | O |
| Tablet computer | O | O | O | O | O |
| Mobile phone | O | O | O | O | O |

TV31) When listening to music, what are the most frequent problems you encounter? You may describe up to 4 problems. Please rate the frequency of the problem by including one of the following numbers at the beginning of your response (1=never, 2=rarely, 3=sometimes, 4=often, 5=always).

Problem #1 _ _ _ _ _ _ _ _ _ _ _ _ _ _ _ _ _ _ _ _ _
Problem #2 _ _ _ _ _ _ _ _ _ _ _ _ _ _ _ _ _ _ _ _ _
Problem #3 _ _ _ _ _ _ _ _ _ _ _ _ _ _ _ _ _ _ _ _ _
Problem #4 _ _ _ _ _ _ _ _ _ _ _ _ _ _ _ _ _ _ _ _ _
