# Supplementary material for: TV listening and hearing aids
Source: PLoS One. 2018 Jun 29;13(6):e0200083. doi: 10.1371/journal.pone.0200083 (PMC6025866; doi:10.1371/journal.pone.0200083)
Supplement: S2 Appendix — (PDF) [file pone.0200083.s002.pdf]

S2 Appendix – Respondents who wear their hearing aids rarely or never when watching TV

The participants who reported wearing their HAs rarely or never when watching TV (non-HA-for-TV users) constituted a small group of 20 participants. Compared with the 240 HA users, they were of similar ages (median of 55 years) but consisted of more male participants (75% in the former vs. 56% in the latter group). They had similar self-reported degrees of HI (32% mild, 47% moderate, 21% severe; Pearson's Chi-squared test, $p = 0.3$) and total HHIA scores (average of 52 vs. 55; Kolmogorov-Smirnov test, $p = 0.62$) as the HA users, but used their HAs less often than these ($p < 0.0001$): None of them used their HAs more than 8 hours per day, and the majority of 55% used them 1 to 4 hours per day. They had slightly lower mean SADL scores than the HA users, 4.0 and 4.4, respectively ($p < 0.05$), with the largest differential group trends on the following two items: They were less affirmative than the HA users on HAs being worth the trouble and affirmed more strongly that wearing HAs made them seem less capable.

In response to the TV questionnaire, the non-HA-for-TV users differed significantly from the HA users with regard to the speakers they used when watching TV. They were 3.5 times more likely to report use of headphones or TV ears (without HAs) than the HA users (45% of non-HA-for-TV users vs. 13% of HA users; Pearson's Chi-squared tests, $p_{BY} = 0.01$) and tended to use the built-in TV speakers less (lower percentage by a factor of 1.5, $p_{BY} = 0.08$). Furthermore, they reported significantly fewer difficulties than the HA users when watching TV (TV item 23) (significant group effect in ANOVA on GLMM using a binomial distribution: $\chi^2(1) = 4.42$, $p = 0.04$, log-odds group difference of 0.64). Also, they were less likely than the HA users to report use of the compensation strategies suggested in TV item 19 when experiencing difficulties with understanding speech on TV [$\chi^2(1) = 4.28$, $p = 0.04$, log-odds group difference of 0.66]. In particular, the non-HA-for-TV users were less likely to turn up the TV volume, turn on CC, or get closer to the TV than the HA users. Finally, they reported less satisfaction with their HAs when watching TV than the HA users (TV item 22) (Pearson's Chi-squared

test, $p = 0.02$). For example, 45% of the non-HA-for-TV users were “slightly satisfied” or “not at all satisfied” with their HAs when watching TV, compared with 15% of the HA users. Thus, their low satisfaction scores with HAs when watching TV mirrored their low usages of HAs when watching TV.
