# Supplementary material for: TV listening and hearing aids
Source: PLoS One. 2018 Jun 29;13(6):e0200083. doi: 10.1371/journal.pone.0200083 (PMC6025866; doi:10.1371/journal.pone.0200083)
Supplement: S3 Appendix — (PDF) [file pone.0200083.s003.pdf]

S3 Appendix – Media listening environment, viewing equipment, and types of shows watched

Media listening environment

In response to item TV2, "How often do you watch TV or video in the following locations?", 88% of the matched non-HA owners and HA users indicated that they watched "often" or "always" at home. With regard to the other locations, the majority of participants answered that they watched "never" or "rarely" at work (84%), on public transport (83%), or in the car (82%). There were no significant differences between the groups (Pearson's Chi-squared tests, $p_{BY} > 0.9$). In response to item TV4, "At home, how often do you watch TV in the following rooms?", the living room was the most popular choice (80% watched there "often" or "always"), followed by the bedroom (58% watched there "sometimes" or "often", while 30% watched there "never" or "rarely"). The majority of participants watched "never" or "rarely" in the dining room (77%) or kitchen (77%) (no significant group differences, $p_{BY} > 0.4$). Furthermore, 40% and 34% specified the size of the room where they watched TV/video most of the time (item TV5) as 13 to 20 m^2 and 20 to 30 m^2, respectively (no significant group difference, $p = 0.84$). 72% indicated that the room had mostly carpeted floor (item TV6) (73% of non-HA owners and 72% of HA users, no significant group difference, $p > 0.9$). The most frequently stated viewing distances (TV7) were 1.5 to 2.4 m (52%) and 2.4 to 3.7 m (35%) (no significant group difference, $p = 0.08$). External background noise was present in the listening environment (TV10) with the following response frequencies: "rarely" (24%), "sometimes" (50%), "often" (20%) (no significant group difference, $p = 0.55$).

Viewing equipment

In addition to the questions about the listening environment, the participants answered three questions about their viewing devices (TV3) and loudspeakers (TV8, TV9). The TV set was the most popular viewing device (85% indicated they used it

"often" or "always"), followed by the PC/laptop (used "often" by 29%, "sometimes by 32%, and "never" or "rarely" by 34%). Most participants watched "never" or "rarely" on a tablet computer (60%) or mobile phone (71%). There were no significant group differences ($p_{BY} > 0.9$). It is interesting to compare these results with the hours of device usage reported by Nielsen [65], who found that adults of ages 18+ watched TV or video 5 hours 10 min per day on a TV set, 13 min per day on a PC, 11 and 14 min per week on a tablet computer and mobile phone, respectively. Thus, our response categories "often" and "always" may reflect usages of several hours per day, the category "sometimes" may reflect usages of several minutes per day, and the category "rarely" may correspond to usages of a few minutes per week. In response to item TV8 ["What type of speakers do you use? (select all that apply)"], 79% of the participants indicated that they used the "speakers in the TV," 33% used "external loudspeakers," and 12% used "headphones or TV ears (without hearing aids)" (no significant group differences, $p_{BY} > 0.9$). Furthermore, 0.7% and 6.3% of the HA users indicated usages of "induction loop systems" and "TV-to-HA streamers", respectively (the percentages were similar in the unmatched group of all 260 HA owners, 2.3% and 5% for induction loops and TV-to-HA streamers, respectively). Although 33% participants indicated they used external loudspeakers, 48% answered the following question TV9, "If you use external loudspeakers, which type to you use?". Since the majority of participants (76%) answered item TV8 about the type of speakers by selecting a single response option, it is possible that they indicated the speakers that they regularly used in response to item TV8 while respondents to item TV9 may also have included participants who used external speakers only occasionally. The percentages in response to this item were 17% for two external speakers, 3.9% for three external speakers, and 13% for a surround sound (five or more loudspeakers). The sum (33%) is consistent with 27% of U.S. households owning an audio/video receiver with surround sound processor in 2015 [56]. 15% of the participants indicated they used soundbars, which is consistent with 16% of U.S. households owning them [56]. There were no significant group differences ($p = 0.85$).

Types of shows watched

With regard to the types of shows watched (item TV11), 61% of the participants watched news broadcasts “often” or “always”, while 43% watched dramas, 37% watched sitcoms, 35% watched documentaries, 33% watched sports, and 18% watched game shows “often” or “always.” The non-HA owners and HA users did not differ in terms of the types of shows watched (Pearson’s Chi-squared tests, $p_{BY} > 0.9$). However, there was a significant effect of gender: male participants watched sports more frequently than female participants ($p_{BY} < 0.0001$; e.g., 45% of male participants vs. 18% of female participants watched sports often or always). In hindsight, the types of shows provided in item TV11 may not have been the best choices. For sake of clarity and generality, it might have been better to use “movies” instead of “drama” and “live shows” instead of “game shows” as in [19]. Furthermore, since the participants could select each of the response options “never,” “rarely,” “sometimes,” etc. for each of the shows, it was not possible to derive a single variable representing the type of show most commonly watched by each participant, which could otherwise have been assessed as a potential predictor of TV problems.

References

19. Liebl T, Weitnauer M. Bessere Sprachverständlichkeit im Fernsehen, speziell für Hörgeschädigte. In: Sammelband 2016, 29th Tonmeistertagung. VDT Int. Conv. Nov. 2016. Köln, Germany.

56. CEA. 17th Annual CE ownership and market potential study. CEA Market Research Report, Consumer Electronics Association (now: Consumer Technology Association); 2015. Arlington, VA.

65. Nielsen. The total audience report Q1, 2015. The Nielsen Company (US), LLC., New York, NY. 2015:p. 8.
