# Supplementary material for: TV listening and hearing aids
Source: PLoS One. 2018 Jun 29;13(6):e0200083. doi: 10.1371/journal.pone.0200083 (PMC6025866; doi:10.1371/journal.pone.0200083)
Supplement: S1 Table — (PDF) [file pone.0200083.s005.pdf]

S1 Table – Demographic and audiologic characteristics of the 515 respondents

Variable	Non-HA owners (*N* = 255)		HA owners (*N* = 260)	
**Age group**		(years)		(years)
	Minimum	50	Minimum	50
	25th percentile	52	25th percentile	52
	Median	55	Median	55
	75th percentile	60	75th percentile	60
	90th percentile	66	90th percentile	65
	Maximum	77	Maximum	83
	NR	4.3%	NR	6.5%
**Gender**		(%)		(%)
	Female	47.8	Female	40.8
	Male	49.0	Male	57.7
	Other	0.4	Other	0.4
	Do not wish to disclose	1.6	Do not wish to disclose	0.8
	NR	1.2	NR	0.4
**Hearing impairment**		(%)		(%)
	Bilateral	85.5	Bilateral	78.5
	Unilateral	14.5	Unilateral	21.2
**Self-reported degree of hearing impairment†**		(%)		(%)
	Mild	38.8	Mild	18.1
	Moderate	50.2	Moderate	60.0
	Severe	11.0	Severe	21.2
	NR	0	NR	0.8
**Mean HHIA scores††**		(score)		(score)
	Total	38.8	Total	55.1
	Social	18.7	Social	26.6
	Emotional	20.1	Emotional	28.5
**Mean global ECHO/SADL scores**		(score)		(score)
	ECHO	3.7	SADL	4.4
**Importance of hearing well when watching TV**		(%)		(%)
	Not at all important	0.4	Not at all important	0
	Slightly important	2.7	Slightly important	1.5
	Moderately important	10.6	Moderately important	13.5
	Very important	49.8	Very important	43.1
	Extremely important	36.1	Extremely important	41.9
	NR	0.4	NR	0

**HA use**				(%)
			Bilateral	58.1
			Unilateral	41.9
**Lifetime HA experience**				(%)
			Less than 6 weeks	4.6
			6 weeks to 11 months	23.8
			1 to 10 years	61.9
			More than 10 years	9.2
			NR	0.4
**Daily HA usage**				(%)
			None	0.4
			Less than 1 hour/day	6.2
			1 to 4 hours/day	22.7
			4 to 8 hours/day	31.9
			8 to 16 hours/day	38.5
			NR	0.4
**HA usage when watching TV**				(%)
			Never	1.2
			Rarely	6.5
			Sometimes	22.7
			Often	28.5
			Always	41.2
**Satisfaction with HAs when watching TV**				(%)
			Not at all satisfied	3.1
			Slightly satisfied	14.6
			Moderately satisfied	41.5
			Very satisfied	33.5
			Extremely satisfied	6.5
			NR	0.8

Demographic and audiologic characteristics of the 515 non-HA owners and HA owners that participated in the study. Results for the TV/media-related items TV12, TV21, and TV22 are also shown.

NR = no response.

†The rating for the worse ear was used.

††HHIA maximum scores for total, social, and emotional were 100, 48, and 52, respectively.
